# Advanced Hepatic Fibrosis in Fatty Liver Disease Linked to Hyperplastic Colonic Polyp

**DOI:** 10.1155/2017/2054871

**Published:** 2017-01-03

**Authors:** Mahmud Mahamid, Omar Abu-Elhija, Tarik Yassin, William Nseir

**Affiliations:** ^1^Internal Medicine Department, Holy Family Hospital, Nazareth, Israel; ^2^Faculty of Medicine in the Galilee, Bar-Ilan University, Safed, Israel; ^3^Internal Medicine Department, The Baruch Padeh Medical Center, Poriya, Israel; ^4^Internal Medicine Department, EMMS the Nazareth Hospital, Nazareth, Israel

## Abstract

*Aim*. Our study aims to determine possible association between biopsy-proven nonalcoholic steatohepatitis (NASH) and hyperplastic polyps (HP) of the colon.* Methods*. A retrospective cohort observational study. All subjects underwent screening colonoscopy within two years. Data were extracted from the patient charts including demographic, anthropometric measurement, vital signs, underlying diseases, medical therapy, laboratory data, results of the liver biopsy with degree of fibrosis and necroinflammatory activity, the colonoscopy report, and the pathological report of the extracted polyp.* Results*. A total of 223 patients were included in our study, 123 patients with biopsy-proven NASH and 100 patients without NASH who served as the control group matched for age. 14 colonic adenomas (11% of patients) were found in the NASH group compared with 16 adenomas (16% of patients) found in the control group (*P* = 0.9). 28 HPs were found in the NASH group (22.7%) compared with only 8 HPs in the control group (8%) (*P* < 0.05). 21 from the 28 (75%) HPs diagnosed in the NASH group were observed in the high degree fibrosis patients (Fibrosis Stages 3 and 4), 6 HPs (21%) were associated with Fibrosis Stages 1 and 2, and single HP (4%) was associated with Fibrosis Stage 0.* Conclusions*. Our study showed an association between biopsy-proven steatohepatitis and the burden of hyperplastic polyp. The severity of hepatic fibrosis may play important role in the increased occurrence of HPs.

## 1. Introduction

Despite being the most common type of polyp detected in the human colon and rectum, relatively little is known about the etiology, natural history, or growth of hyperplastic polyps (HP) [1, 2].

HPs are considered benign lesions that have little or no malignant potential. However, recent studies have suggested that HP may either lie in the classic adenoma-carcinoma pathway [3, 4].

Lifestyle and dietary risk factors are the most common risk factors for development of HP according to epidemiological studies; other risk factors include alcohol consumption, cigarette smoking, obesity, metabolic syndrome, and fiber intake [5, 6].

Nonalcoholic fatty liver disease (NAFLD) is emerging condition and constituted an important public health problem across the globe. Fatty liver disease can present as simple hepatic steatosis that may progress to nonalcoholic steatohepatitis (NASH), fibrosis, cirrhosis, and hepatocellular carcinoma (HCC).

NAFLD is the most common cause of incidental elevated liver enzymes in the developed world. The prevalence of NASH in Europe and USA ranges from 14 to 20%; this increase in the prevalence is related directly to obesity epidemically seen in these populations [7].

Diabetes mellitus (DM), obesity, and hyperlipidemia are common components of the metabolic syndrome (MS), which is frequently associated with the NAFLD. Therefore, NAFLD is the hepatic manifestation of MS [8].

The pathogenesis of NAFLD is not clearly elucidated, but accumulating data suggest that insulin resistance (IR), oxidative stress, lipotoxicity, intestinal endotoxins, and bacterial translocation which all related to the MS play a crucial role in the pathogenesis of steatosis, steatohepatitis, and fibrosis [9].

Although many studies have proposed that MS is a risk factor for colorectal cancer (CRC), colonic adenomas, hyperplastic polyps, and NAFLD [10], there have been no studies that investigate an association of hyperplastic polyps and NAFLD. Therefore, we investigated whether a relationship of HP to NAFLD could be determined.

## 2. Methods

A retrospective cohort observational study was conducted at Division of Internal Medicine of the Holy Family Hospital, Nazareth, Israel, between April 2013 and April 2015 on patients who underwent screening colonoscopy.

The study was approved by the local ethics committee. The data were coded to keep anonymity of the patients. Informed consent was waived because of the noninterventional study design.

### 2.1. Patient Selection and Data Collection

The study population of this study consisted of adult patients with biopsy-proven NASH who underwent a screening colonoscopy in the past two years. Subjects were followed up at the Internal Medicine Department of the Holy Family Hospital (HFH). Patients were included only if they had underwent a screening colonoscopy at our facility within 2 years and underwent liver biopsy in the past 5 years.

### 2.2. Exclusion Criteria Included

NASH patients without biopsy performed to confirm the diagnosis and patients with other liver diseases, with known colonic disease including inflammatory bowel disease and polyposis syndrome, with history of total or segmental colectomy, with family or personal history of colonic cancer or colonic polyps, and with sessile serrated adenomas. The control group consisted with non-NASH patients, matched in age.

Data were obtained from the medical charts of all enrolled patients and from family physician especially in case that there was any missing data for study purposes. The following information was extracted from patient's charts: demographic, anthropometric measurement, vital signs, underlying diseases, medical therapy, laboratory data, and result of the liver biopsy with degree of fibrosis and necroinflammatory activity, the colonoscopy report, and the pathological report of the extracted polyp.

### 2.3. Measurements

The body mass index (BMI) for all patients was calculated in kg/m^2^. A single expert hepatopathologist reviewed all liver biopsies and utilized the scoring system established by Brunt and colleagues [11] for grading and staging of steatohepatitis (staging of fibrosis is as follows: Stage 0, no fibrosis, Stage 1, zone 3 perisinusoidal fibrosis, Stage 2, as above with portal fibrosis, Stage 3, as above with bridging fibrosis, and Stage 4, cirrhosis).

### 2.4. Colonoscopy

Each patient underwent colonoscopy after completing a bowel preparation with 4 liter polyethylene glycol lavage solution; colonoscopies were performed by one of the five staff gastroenterologists at HFH; each colonoscopy report was examined for the present of polyps, polyp location, polyp size, number of polyps, and histology; the location of polyp was divided into cecum, ascending, hepatic flexure, transverse, splenic flexure, descending, sigmoid, and rectal.

### 2.5. Statistical Analysis

Data were analyzed using SPSS version 19 (IBM SPSS, Chicago, IL, USA). Continuous variables are expressed as the mean ± standard deviation. The Chi-square test was used to test differences in categorical variables between the cases and controls, and analysis of variance (ANOVA) or Student's *t*-test was used for comparisons of continuous variables. Spearman rank correlation and univariate regression analysis were used to determine the strength of the relationship between NAFLD and hyperplastic polyps after adjusting for independent variables previously known to be associated with HP, namely, age, gender, BMI, and diabetes mellitus. A multiple logistic regression analysis was done to determine the association between the different risk factors for HP. A significant level of <0.05 was used in this test.

## 3. Results

A total of 223 patients who underwent screening colonoscopy were included in our study, 123 patients with biopsy-proven NASH and 100 patients without NASH who served as the control group.  Table 1 summarizes the clinical feature of the two groups, those with and without NAFLD.

Among the NASH group the 72 patients (86%) were males; the mean age was 41 ± 13, the mean body mass index was 25.3 ± 4.7, patients who meet the criteria of metabolic syndrome were 88 patients (72%), and the mean C-reactive protein level was 1.1 ± 0.7.

Regarding the polyps prevalence, 14 adenomas (11% of patients) were found in the NASH group compared with 16 adenomas (16% of patients) found in the control group (*P* = 0.9). No significant differences were found when adjusting for known confounders to include age and BMI (*P* = 0.34). There was no significant increase in the risk for colonic adenomas when comparing individual component for MS and MS alone adenoma.

The location, size, number, morphology, and degree of dysplasia of the adenomas were similar between the two groups.

The prevalence of HP was statistically significant higher among the NASH group; 28 HPs were found in the NASH group (22.7%) compared with only 8 HPs in the control group (8%). The size and morphology of the polyps were similar but the location differs between the groups, all HPs in the NASH group were located in the left colon, and 2/8(25%) of HPs in the control group were located in the right colon.

The degree of liver fibrosis among the NASH group according to the liver biopsy was as follows: Fibrosis Stage 0 on 18 patients (15%), Fibrosis Stage 1 on 29 patients (23%), Fibrosis Stage 2 on 32 patients (26%), Fibrosis Stage 3 on 37 patients (30%), and Fibrosis Stage 4 on 7 patients (6%).

It was a significant correlation between the degree of fibrosis and the prevalence of HPs as 21 from the 28 (75%) HPs diagnosed in the NASH group were observed with high degree of fibrosis (Fibrosis Stages 3 and 4), six HPs (21%) were associated with Fibrosis Stages 1 and 2, and a single HP (4%) was associated with Fibrosis Stage 0.

The univariate and multivariate analyses of the risk for HPs are shown in Table 2. From the logistic regression analysis, older age (>50), male sex, current smoking, high CRP levels, NASH, and advanced fibrosis were associated with increased risk for HP (Table 2).

Regarding the relationship of MS and NASH status with the presence of HP (Table 3), compared with MS negative/NASH negative group, the MS negative/NASH positive group and the MS positive/NASH positive group were significantly associated with increased risk for HPs (OR 1.3; 95% CI, 1.02–2.13 and OR 1.48; 95% CI, 1.05–2.04, resp.).

## 4. Discussion

This is the first study to evaluate the relationship between biopsy-proven steatohepatitis and colonic hyperplastic polyps; the results of this study demonstrate that NASH was significantly associated with an increased frequency of colonic hyperplastic polyps. Moreover, the frequency of HPs was correlated with the degree of liver fibrosis, as in patients with advanced fibrosis the possibility of having HP is higher than that in those with early or no hepatic fibrosis. Moreover, NASH and advanced hepatic fibrosis are associated with HPs independently by age, sex, smoking status, and metabolic syndrome.

Interestingly, we found a correlation between the occurrence of HPs and increased levels of C-reactive protein, as an independent predictor of HPs [12, 13].

A previous study reports an association between metabolic syndrome and colorectal cancer or/and colorectal adenomatous polyps [14, 15]. Moreover, a large population based study from Korea comparing the relationship between ultrasound diagnosed NAFLD and colorectal adenomas found a direct association with NAFLD and colonic adenomas [16]. Other studies from USA found similar association between biopsy-confirmed NAFLD and the prevalence of colonic adenomas [17]. Currently, patients with more than 3 colonic adenomas or any adenoma >10 mm are subject to colonoscopic screening every 3 years [18]. Thus, patients with NAFLD may need more stringent endoscopic follow-up.

NASH/NAFLD is commonly referred to as the hepatic manifestation of MS; patients with MS have been shown to have higher risks of colorectal HPs, as illustrated by several studies [19, 20]. The mechanism that joins the two entities is most likely linked to insulin resistance (IR) that may play a distinct role in development of HPs [20, 21].

Adiponectin is an adipokine that is found in decreased concentrations not only in those who are obese and have diabetes and IR (which all is important component of the MS) but also in NAFLD/NASH patients; decreased adiponectin levels lead to increased insulin levels due to IR and increased insulin growth factor-1 (IGF-1) [22–24]. Adiponectin also directly inhibits tumor necrosis factor-alpha (TNF-*a*), which plays a role in tumor cell proliferation and angiogenesis (recent studies have shown that HPs may lie in the classic adenoma-carcinoma sequences and have demonstrate special molecular changes and genetic mutations which correlated with hyperplastic polyps or/and serrated colonic polyps which represent mixed feature of colonic adenomas and HPs) [25–27].

Low adiponectin levels are inversely related to colonic tumors stage and predict cancer recurrence [28, 29]. Insulin binds to IGF-1 receptors and plays an important role in cell proliferation, apoptosis, and increased production of vascular endothelial growth factor, an angiogenetic factor that supports tumor growth and may play a role in the development and growth of HPs [30].

In NAFLD, the hepatic manifestation of the MS and plasma inflammatory biomarkers are increased leading to chronic and systemic low-grade inflammatory state, mediators from the liver, including reactive oxygen species (ROS), TNF-*a*, IL-6, and PAI-1, and other proinflammatory cytokines which were positively associated with the prevalence of colorectal cancer, colonic adenomas, and most probably HPs [31]. According to our finding the combination of both NASH and MS increases the frequency of HPs due to the involvement of all the previously suggested factors.

Other interesting findings of our study are the direct correlation between hepatic fibrosis and the prevalence of HPs; HPs are more common in advanced hepatic fibrosis and cirrhotic patients compared with early or no hepatic fibrosis; further studies are required to confirm this hypothesis and to elucidate possible mechanism responsible for the phenomenon.

Our study had several limitations; the retrospective design of the study makes it difficult to infer causality between NASH and risk for HPs. Second, there may have been a selection bias, as subjects were recruited from patients who visit the hospital (HFH) for health examination, and thus they were more concerned about their health status. Third, the population size was small to accurately reflect some known risk factors to be associated with HP as ethnicity and family history. Fourth, subjects did not undergo any molecular pathology diagnostics [32].

In conclusion, our study is the first to show an association between biopsy-proven steatohepatitis and the burden of hyperplastic polyp; the severity of hepatic fibrosis may play important role in the increase frequency of HP in those patients; further prospective studies are required to confirm the hypothesis that NASH especially with advance fibrosis may cause colorectal hyperplastic polyp.

## Figures and Tables

**Table 1:** Demographic, laboratory, and clinical data comparing NASH group and control group.

Characteristic	Case *n* = 123 (%)	Control *n* = 100 (%)	*P* value
Age (years)	41 ± 13	42 ± 12	NS
Male sex	72 (86%)	43 (43%)	<0.05
BMI (kg/m^2^)	25 ± 4.7	24.7 ± 3.2	NS
Metabolic syndrome	88 (72%)	57 (57%)	NS
C-reactive protein	1.1 ± 0.7	0.4 ± 0.8	<0.05
Ethnicity (Arabs)	95 (77%)	72 (72%)	NS
Diabetes mellitus	37 (30%)	32 (32%)	NS
Hypertension	42 (34%)	39 (39%)	NS
Hyperlipidemia	49 (40%)	38 (38%)	NS
Smoking	8 (15%)	11 (22%)	NS

**Table 2:** Multivariate analysis for the risk for hyperplastic polyp by gender, age, smoking, CRP level, prevalence of NASH, and the degree of liver fibrosis.

Variable	OR (95% CI)	*P* value
Male gender	1.80 (1.02–1.66)	<0.001
Age > 50	2.24 (1.91–2.13)	0.003
CRP > 2 mg/L	1.47 (1.14–2.18)	0.027
Current smoking	1.35 (1.05–1.67)	0.022
Prevalence of NASH	1.69 (1.36–198)	<0.001
Fibrosis > stage 2	1.82 (1.23–1.76)	<0.001

OR, odds ratio; CI, confidence interval; CRP, C-reactive protein.

**Table 3:** Univariate and multivariate analysis of the risk for hyperplastic polyps by NASH and metabolic syndrome.

Variable	Univariate analysis; OR (95% CI)	Univariate analysis; *P* value	Multivariate analysis; OR (95% CI)	Multivariate analysis; *P* value
MS−/NASH−	1	—	1	—
MS+/NASH−	1.48 (0.94–2.34)	0.193	1.31 (1.01–1.77)	0.0568
MS−/NASH+	1.62 (1.27–2.03)	<0.001	1.30 (1.02–2.13)	0.034
MS+/NASH+	1.96 (1.47–2.72)	<0.001	1.48 (1.05–2.04)	0.018

OR, odds ratio; CI, confidence interval; NASH, nonalcoholic steatohepatitis; MS, metabolic syndrome.
