# Corrigendum to “Advanced Hepatic Fibrosis in Fatty Liver Disease Linked to Hyperplastic Colonic Polyp”

**DOI:** 10.1155/2017/9042568

**Published:** 2017-12-28

**Authors:** Mahmud Mahamid, Omar Abu-Elhija, Tarik Yassin, William Nseir

**Affiliations:** ^1^Internal Medicine Department, Holy Family Hospital, Nazareth, Israel; ^2^Faculty of Medicine in the Galilee, Bar-Ilan University, Safed, Israel; ^3^Internal Medicine Department, The Baruch Padeh Medical Center, Poriya, Israel; ^4^Internal Medicine Department, EMMS Nazareth Hospital, Nazareth, Israel

The article titled “Advanced Hepatic Fibrosis in Fatty Liver Disease Linked to Hyperplastic Colonic Polyp” [1] uses the same cohort and study population as Mahamid et al., “Association between Fatty Liver Disease and Hyperplastic Colonic Polyp,” The Israel Medicine Association Journal, Volume 19, Number 2, February 2017 [2]. This was raised by Ribaldone et al. [3]. The authors apologize for not citing this article and not discussing its relationship with their work.

The two articles are similar regarding the study population, but there is a significant difference between them regarding the main findings; the first article published at IMAJ showed the association between the two diseases. The second published at CJGH showed novel and interesting association between the degree of fibrosis in steatohepatitis (not the disease itself) and hyperplastic polyp.
